# Health Promotion and Disease Prevention Strategies in Older Adults with Intellectual and Developmental Disabilities

**DOI:** 10.3389/fpubh.2014.00031

**Published:** 2014-04-14

**Authors:** Eli Carmeli, Bita Imam

**Affiliations:** ^1^Department of Physical Therapy, Faculty of Social Welfare and Health Sciences, University of Haifa, Haifa, Israel; ^2^Rehabilitation Research Lab, GF Strong Rehab Centre, University of British Columbia, Vancouver, BC, Canada

**Keywords:** health promotion, older adults, intellectual disability, physical activity, nutrition, social

## Abstract

The rapid growth in the number of individuals living with intellectual and developmental disabilities (IDD) along with their increased longevity present challenges to those concerned about health and well-being of this unique population. While much is known about health promotion and disease prevention in the general geriatric population, far less is known about those in older adults with IDD. Effective and efficient health promotion and disease prevention strategies need to be developed and implemented for improving the health and quality of life of older adults living with IDD. This is considered to be challenging given the continued shrinkage in the overall health care and welfare system services due to the cut in the governmental budget in some of the western countries. The ideal health promotion and disease prevention strategies for older adults with IDD should be tailored to the individuals’ health risks, address primary and secondary disease prevention, and prevent avoidable impairments that cause premature institutionalization. Domains of intervention should include cognitive, mental and physical health, accommodations, workplace considerations, assistive technology, recreational activities, and nutrition.

## Introduction

Older adults with intellectual and developmental disabilities (IDD) are made up with several characteristics and uniquenesses:

- The majority of older adults with IDD live with their family or in a community home, while others live in private or governmental hostels or residential care centers (1, 2).
- Over the next two–three decades, more adults with IDD will be living longer into their 70s and 80s (3).
- Older adults with IDD are exposed to a range of health problems, and a rapid functional and cognitive decline.
- Older adults with IDD deprived some social support such as financial support, accommodation, health care regulation (i.e., regular annual physical and mental health checks), and lifestyle (nutrition, physical exercises, safety). They require services by adequately trained and educated skilled staffs and caregivers (4).
- Some individuals with IDD such as adults with Down syndrome (DS) are more likely to develop early-onset dementia and consequently are more likely to die at a younger age (5).

Health promotion and disease prevention strategies are lacking, particularly related to (a) early detection of co-morbidities (6); (b) mental health issues, communication impairments, and poor environments; (c) social problems that challenge the society (7); and (d) early-onset of aging that requires early detection and intervention (8).

## Policies in Relation to Longevity

In 2001, the World Health Organization (WHO) and the International Association for the Scientific Study of Intellectual Disability (IASSID) introduced comprehensive guidelines and policies in relation to older adults with IDD (9). These guidelines are presented briefly below:

- Since the life expectancy of individuals with IDD is somewhat shorter than the general population, along with a poor mental and physical health status, planning ahead “*cannot be based solely on chronological age cut-offs*.”
- The overall support system should be throughout the life-span with the aim of making the most of function and independence.
- Efforts must be done to enhance the quality of life of older adults with IDD, to promote fairness and equality of opportunities, and to increase their participation in the society.
- The policy objectives require the development of general infrastructures and funding obligations to provide access to services, including skilled nursing facility, transportation, job opportunities, and professional training.
- Resources and information (internet, library, journals, and knowledge of expertise) should be available to clinicians, therapists, families, caregivers, and support groups working with this population.

## Mental Health Promotion and Prevention

There is a documented co-existence between older adults with IDD and mental health problems, such as affective disorders, aggressive behavior, psychotic conditions, and eating disorders (10, 11). Individuals with IDD are more likely to develop “pre-aging phenomenon” such as dementia and anxiety (12), and to develop poor balance, bad posture, and gait difficulties, and consequently will require closer care.

Health promotion is needed to help older adults with IDD and their caregivers to live a fulfilling life with a reduced level of insecurity, stress, and anxiety. Mental health promotion strategies include formal and informal support such as welfare services, governmental offices, public and private agencies, voluntary organizations, charitable associations, family caregivers, and relatives.

Many older adults with IDD are living with family and may live longer than their parents. Therefore, additional future burden will fall on external services (13), but whether these services can meet the demands are yet to be seen. Previous reports by WHO have indicated that “*local authorities in many western countries are failing to meet these demands*” (14). Suggested solutions to administer and treat mental health problems are:

- Holistic assessment that aims to identify both physical health problems and environmental factors that might contribute to the etiology of mental health problems is fundamental (15–17).
- Paper work, governmental regulations, and policy of documentation, which aim to provide standardized guidelines must be developed.
- Appropriate living arrangements must be made. Older adults with IDD should have their own place to live at and a choice with whom they want to live.
- Providing appropriate job opportunities is significant. Older adults with IDD are one of the most deprived population in the society in terms of job opportunities and have the lowest employment rate (18).
- Providing training to paid mental health care staff and non-paid care givers can give way a better competence in management of mental problems which is also important for improving the quality of life and reducing the prevalence of those mental disorders (19).
- For a program to be effective, there should be a “mutual approach” that is client-centered. The program should focus on prevention, and be practical and sustainable (20). The “multi factor approach” should include also medications (21, 22), psychotherapeutic (23, 24), psycho sensory-motor therapy (25), environmental management (26), social support (27), and family education (28).

## Physical Health Promotion and Disease Prevention

There is plenty of evidence that shows cardiovascular–respiratory disease as a common cause of death among older adults with IDD, as it is in the general population; however variations in prevalence and severity depend on the level, lifestyle, and functional significance of the person with IDD (29, 30). Older adults with IDD are at a high risk for developing digestive problems, including *Helicobacter pylori*, gastroesophageal reflux disease, and constipation (31). In addition, poor nutrition, lack of physical activity (PA), and having a sedentary lifestyle lead to occurrence of primary sarcopenia and osteoporosis type II and obesity.

## Management Strategies – Focusing on Dementia

In the last decade, healthy aging in people with IDD has received an increased attention in the literature (32–34). The overall consensus among researchers is that over the life-span, adults with IDD should maintain the same way of preventive health services as offered to the general population (35). As longevity of people with IDD increases, it is likely that there will be a consequent increase in the number of these people suffering from age-related diseases such as dementia (36). Dementia is defined as a syndrome due to the disease of the brain, progressive in nature, in which there is disturbance of higher cognitive functions, including memory, thinking, orientation, comprehension, calculation, learning capacity, language, and judgment (37). The increased prevalence of dementia amongst older adults with IDD, in conjunction with other health problems, presents significant challenges to formal and informal IDD services, primary healthcare, their extended families, and their local communities. Moreover, since there are a variety of different tools for the assessment of dementia in IDD, the health system must reach consensus regarding what approach or instrument would be the best to use. Establishing this would improve the quality of assessment in clinical practice and allow research to have more informative value (38).

Secondary prevention that focuses on IDD people with high risk of developing dementia such as DS is crucial to yield a beneficial effect. Such approach requires initiating screening, observation, followed by a clinical examination and pharmacological and psychological interventions as early as possible (39).

Social problem-solving programs have shown success in reducing aggressive behaviors among individuals with intellectual disabilities in clinical settings, and should be adapted for health promotion in community settings (40).

## Physical Health Promotion, Prevention, and Down Syndrome

The most common cause of IDD is DS (or trisomy 21). Persons with DS are inclined to a shorter life-span. The reason for the unrelenting shorter life-span in the DS population, compared to many other developmental disability populations, is thought to be due to an accelerated aging process, which is manifested by an increased risk for heart problems, metabolic syndrome, cataracts, hearing loss, osteopenia/porosis, hypothyroidism, and Alzheimer’s disease. Moreover, individuals with DS have unique physiological and physical characteristics, such as muscle hypotonicity, joint hypermobility, and increased tendency for obesity (41, 42).

Previous studies have shown that isokinetic measurements of the muscles of the lower limbs and the scores on the functional balance assessments such as “time-up-and-go” were lower in persons with DS (43, 44). Muscle weakness in persons with DS is associated with impairments in aerobic capacity and physical function (45).

Osteoporosis affects both females and males with IDD, and it can have detrimental effects (46). Estrogen deficiency appears to be a major factor in the pathogenesis of osteoporosis in both genders (47). However, mortality after a hip fracture, one of the major complications of osteoporosis, is more common in males than in females (48).

Some of the risk factors for low bone mineral density (BMD) in older adults with DS include long use of anti-seizure/epileptic drugs (49), low calcium and vitamin D intake, lack of physical exercise, hypogonadism, hypothyroidism, and secondary hyperparathyroidism. Consumption of at least 400 IU vitamin D orally a day, and at least 1,000 mg/day of calcium (50), antioxidants (51), and phytochemicals (52) increases capacity for physical exercises (53).

Obesity is a significant health problem for people with IDD, with a higher prevalence among females and individuals with DS (54). Multiple factors involve or cause obesity, including: (a) extrinsic factors such as poor diet and sedentary lifestyle, and (b) intrinsic factors such as genes that cause predisposition to excessive weight and obesity (55). Obesity leads to a higher risk of developing chronic conditions, such as diabetes, hyperlipidemia, hypertension, cardiopulmonary disease, and Alzheimer’s disease. These health risks could be developed earlier in life presenting early-onset of aging.

Healthcare services have predominantly focused on the primary disability rather than on primary prevention or reduction of secondary health conditions. As health promotion enables people to gain control over their lives, it is essential to address the obesity health concerns for individuals with IDD. The prevalence of obesity differs by living arrangement, with institutional residents having the lowest prevalence and people living in their own home the highest.

## Assistive Technology

Assistive technology (AT) is any device that helps a person with IDD accomplish their daily activities; compensates for functional limitations; affords an opportunity for learning, independence, mobility, productivity, cooperation, or communication environmental control; lowers the risk of secondary conditions; allows caregivers to provide assistance easier; and forestall the need for nursing home care. Individuals with IDD face declines in health and function, and therefore have a greater need for a form of AT.

Assistive technology includes “low-tech” (i.e., simple to use such as walkers), or “high-tech” (i.e., computerized, such as Hoyer/Leverage) devices. Welfare and rehabilitation services must consider AT for any older adult with IDD. That means that for any person receiving special health or rehabilitation services, the professional team must ask if there is a device that will “maintain or improve functional capabilities” of the individual. People with intellectual disabilities should be introduced to AT as early as possible. Once the need for AT is identified, a qualified evaluator (generally a physical or occupational therapist, a speech language pathologist, or an optometrist) must complete an assistive and augmentative technology evaluation (56). Once the AT is acquired, training must be provided. The AT device should be available for use throughout the day and in natural settings, including residential care center, work, and recreation. AT device should be flexible and customized to accommodate the unique abilities of each person with IDD.

In order to facilitate AT uptake, attention should be given to the following:

- AT should help with planning, execution, attention, and memory (e.g., such as assistive listening devices, assistive alerting devices, and assistive signaling devices to maximize the person’s hearing efficiency, enhancing communication, and fostering independence).
- AT should monitor health (e.g., tele-health/medicine/care).
- AT should monitor mobility (e.g., wheelchairs, sliding board, Hoyer lift, cushing). Outdoor spaces also need to be designed for accessibility.
- Watching safety (alert, alarm, and security systems).
- AT should assist with ADLs (e.g., personal hygiene) and IADs (e.g., shopping, telephone use, travel in community, housekeeping, and preparing meals).
- Managing the physical environment and access (curbs, thresholds, stairs, sidewalks gratings, obstructions, narrow passages) is important. Physical access includes accessible routes, curb ramps, parking and passenger loading zones, elevators, signage, and entrances.
- Access to argumentative technology computers, handheld devices, board sign, and picture communication systems should be made available.
- ATs should facilitate sensory limitations, such as hearing difficulties or deafness (e.g., equipping with hearing aids) and vision difficulties or blindness (e.g., by improving vision glasses and equipping with a magnifier TV screen).

## Nutrition

Enormous body of evidence shows that a healthy diet could improve individual’s quality of life and abate existing secondary conditions such as fatigue, weight problems, and constipation. Poor diet is related to many chronic conditions. Dietitians, qualified nutrition professional, as well as personal assistants who plan and prepare meals for adults with IDD should adopt a structured approach to meal planning and preparation, as exemplified by the Minimum Standards of Care proposed by Montana Disability and Health Program (57). The Standards of Care emphasizes that three points that must be addressed: (1) *providing health-promoting food and nutrition supports*; (2) *providing information, knowledgeable encouragement, and positive social/instrumental support (assistance in grocery shopping, cooking, etc.) to help individuals make good food choices; and* (3) *supporting participation in activities that encourage healthy eating and PA*. In order to ensure that older adults with IDD receive the proper diet, the nutrition health promotion must include four levels of standards: (1) must meet the safety standards (stored and prepared); (2) nutrition must be adequate in terms of “health” (‘low LDL cholesterol, and saturated and trans fats; limited simple sugars and salt; more plant proteins such as beans, nuts, grains and fewer and leaner animal proteins (meat); limited candy, sodas, desserts, processed meats, and salty snacks such as chips; multiple vitamin/mineral supplement’ (58); ‘little or no alcohol, variation (fruits, vegetables, and whole grains)), (3) the diet should address the specific health or medical needs, and (4) the diet should respect the individual’s preferences and must be culturally appropriate. Recent meta-analysis shows that adherence to a Mediterranean diet can significantly decrease the risk of overall mortality as well as mortality related to cardiovascular diseases, cancer, Parkinson’s and Alzheimer’s disease’ (59). Nutrition management should have the following components:

- A qualified nutrition professional should review food menus periodically.
- Three meals and healthy snacks should be offered at appropriate times each day, and served in a pleasant atmosphere.
- Individualized diet should be provided. Some individuals need a special diet in order to be adequately nourished.
- The food and fluid intake of each individual should be documented and followed up on.
- Diet and exercise go hand in hand; therefore it is necessary to support older adults with IDD to engage in low to moderately intense PA.
- As energy requirements decrease, the protein density of the diet should be greater for both males and females (i.e., more protein containing foods such as lean meat, milk and dairy foods, eggs, and pulses should be eaten). Recommended intake of protein per day in adults with IDD over 50 years old is 0.8–1.2 g/1 kg of body weight (see Table 1).

**Table 1:** **Estimated energy requirement (EER) according to age**.

Age (years)	Estimated energy requirement for males (kcals/day)	Estimated energy requirement for females (kcals/day)
51–59	2200	1800
60–64	2100	1800
65–74	2000	1800
75+	2000	1800

*EER equations are from the Institute of Medicine. Dietary reference intakes for energy, carbohydrate, fiber, fat, fatty acids, cholesterol, protein, and amino acids. Washington (DC): The National Academies Press (2002)*.

## Physical Activity

The health, age, cognitive ability, and the well-being of older adults with IDD put them at particular high risk for low levels of PA (60, 61). The United States Surgeon General stated that, “*Regular physical activity can help people with chronic and disabling conditions to improve their stamina, muscle strength, psychological well-being, and quality of life by increasing the ability to perform activities of daily life*.” Based on the literature review, individuals with IDD can directly benefit from a meaningful PA program, or indirectly that can benefit from PA if educational plans are provided to their care givers (62).

Practical recommendations for physical activity should include:

- Low to moderate-intensity activities such as walking, biking, or swimming.
- Two and a half hours and 30 min (150 min) of low to moderate-intensity aerobic activity, every week, and muscle-strengthening activities on two or more days a week that work all major muscle groups (legs, hips, back, abdomen, chest, shoulders, and arms) should be done.
- PA should start slowly and gradually increase the intensity (~ 5% every other week).
- The activities need to be enjoyable, fun, and involve social interaction.
- Participants should be involved in activity selection and decision making.
- Activities need to be age-appropriate.
- Parents, siblings, and other care providers can act as role models.
- Be prepared to modify activities to accommodate all abilities.
- Exercise classes should modify to the individual’s needs (for beginners start from a very basic level of fitness to being able to complete 40–50 min of continuous exercise). Exercises mostly should include balance and coordination activities involving endurance, flexibility, and strength training.
- Group exercises provide peer support and help to enhance motivation, attendance, and compliance. T-shirts or caps can be made for participants to give them a sense of group identity. Music, dancing, and games should also be integrated into the activities.
- Some PA such as walking and gardening is better than none at all (63).
- Monitor progress.
- Both diet and PA play critical roles in controlling weight and maintaining good health.

## Work Opportunities to Promote Health

Older adults with IDD are amongst those in society with the lowest employment rate and often experience employment difficulties. Less than 10% of this population in western countries are employed (64). Moreover, in many countries, reports on employment rate are not available and the real work opportunities could be even worse. The limited opportunities for employment for this population may be attributed to the lack of understanding or acceptance of the behavior of a person with IDD, as well as a lack of expertise regarding behavior management and technical barriers. Creating employment opportunities for adults with IDD require: (1) teaching and training workers with IDD; (2) appropriate supports with integrated employment settings; and (3) regulations on workers’ safety and health, and (4) funding.

Training courses should fit the individual’s skills, such as hands-on activities that encourage practice and do not require reading skills. Topics should also include how workers with IDD can identify and protect themselves from workplace hazards, what to do in an emergency, and how to speak up about a safety problem at work. Supportive environment should include information on how to hire workers with IDD, on-job training, and how to reduce job stress, work-related musculoskeletal disorders, and even infectious disease risks.

Regulations regarding health hazards, abuse, and discrimination (promotion, transfer, or access to training) should be provided and innovative ways of obtaining funding, including non-competitive grants awarded for inventive and resourceful projects should be considered.

## Recreational Opportunities

Recreation/leisure activities are currently given low priority as an area in which support and assistance are often required. Many people with IDD are still limited to segregated recreation and leisure choices. But recreation and leisure activities are critical dimensions of the quality of life for older adults with IDD. Leisure and recreational activities provide opportunities for having fun, meeting new friends, participating in neighborhood/community, and developing skills and competencies.

## Social Opportunities

A rise in life expectancy has created a shift in socio demographics leading to an aging IDD population. Today, our society is faced with an ever growing elderly population with IDD with increasing social expectations and demands. However, many older individuals with IDD face social isolation and they are deprived from social engagements due to physical and medical constrains and lack of adjusted services. For IDD individuals to enjoy “successful aging” in which there is an increase, or at least maintain, of well-being and life satisfaction, an active engagement in life must be addressed, along with low probability of disease and disease-related disability and a high level of physical and cognitive function. Social opportunities is a combination of social activity (i.e., shopping), productive activity (i.e., working), interpersonal relationships, participation in leisure activities (i.e., gardening; outdoor sport activities), and belonging to support groups. All of these represent forms of engagement.

Putting all the above into a practical setting, we need to consider older adults with IDD, living in the broader community who are less dependent in their homes or residential care centers, have friendly and a safer environment accessibility without stigmatization for daily functioning and navigation, including the availability of emotional support and physical assistance, and provide value regardless of compensation to the individual (i.e., paid employment).

## Key Points to Consider

The key points apply a medical and social perspective to IDD health. It focuses on health promotion, quality of life, emphasizing the outcomes and not the activity. Another basic premise of the key points is that the health promotion and disease prevention relate to community support. Therefore, improving the quality of life for individuals with IDD depends also on their community, and that the two are inseparable. Planning and implementing comprehensive interventions require reinforcing factors and a joint effort among other health professionals and organizations, policy makers, volunteers, community officials, community leaders including members of welfare ministry that relates to IDD population. Supporting the families and caregivers is crucial, and there are a number of options such as focus and support groups, face-to-face instruction or education, in-service, and so forth that they can be used individually or in combination.

### Principles of health promotion and disease prevention

- Early detection and accurate bio-socio-psycho model diagnosis, identifying impairments and functional decline, setting health priorities.
- Identifying certain concerns, needs, and expectations also of the paid (i.e., therapists) and unpaid care givers (family).
- Determining variety or option of interventions, and defining the optimal outcome. Look for the issues, barriers, and risk factors that might cause or influence the outcomes.
- Evaluating the success or failure and the impact of the intervention.
- Keep integrity in paperwork reports and documents (be precise and be honest).
- Developing and increasing access to health, social, leisure, job, transportation, and community services.
- Maintain personal and professional ethics all time.

### Practical recommendations

- Early detection by health check (fecal occult blood, bone density, cervical/breast/prostate cancer).
- Promotes the engagement in PA, outdoor recreational activities, and social opportunities.
- Support for healthy behavior in life style: personal hygiene, balanced diet, avoid smoking and alcohol.
- Social and recreational resources should be constructed. Young volunteers between the ages of 15 and 21 years old should be recruited from neighboring schools to increase function and fun and to socialize with the individual and their families.
- Multisensory environment such as Snoezelen using lighting effects, color, sounds, music, scents, etc. relaxing atmosphere should be provided.

## Conclusion

Health promotion and disease prevention strategies for older adults with IDD should be modified to the individuals’ health status, social needs and abilities, and psychological concerns. Areas of intervention should include mental and physical health, housing, work opportunities, adapted AT, physical exercises, recreational activities, leisure time, and diet monitoring.

## Conflict of Interest Statement

The authors declare that the research was conducted in the absence of any commercial or financial relationships that could be construed as a potential conflict of interest.
